# Evaluation of real‐life clinical outcomes in Australian youth with type 1 diabetes on hybrid closed‐loop therapy: A retrospective study

**DOI:** 10.1111/jpc.16043

**Published:** 2022-05-31

**Authors:** Sathyakala Vijayanand, Paul G Stevenson, Elizabeth Broad, Elizabeth A Davis, Craig E Taplin, Timothy W Jones, Mary B Abraham

**Affiliations:** ^1^ Department of Endocrinology and Diabetes Perth Children's Hospital Perth Western Australia Australia; ^2^ Telethon Kids Institute University of Western Australia Perth Western Australia Australia; ^3^ Division of Paediatrics, within the Medical School The University of Western Australia Perth Western Australia Australia

**Keywords:** clinical outcomes, hybrid closed loop, real‐life experience

## Abstract

**Aim:**

To determine the clinical outcomes and evaluate the perspectives of children with Type 1 diabetes (T1D) and their parents managing their child on hybrid closed‐loop (HCL) therapy.

**Methods:**

Children with T1D on HCL attending a tertiary diabetes centre between April 2019 and July 2021 were included. A retrospective analysis of glycaemic data was conducted to determine the clinical outcomes. Time spent in closed loop, time in target glucose range (TIR 3.9–10 mmol/L), hypoglycaemia and hyperglycaemia were collected at baseline, 4 weeks, 3 and 6 months post‐HCL. User experience was assessed by questionnaires administered to parents of children with T1D.

**Results:**

Seventy‐one children, mean (SD) age of 12.2 (3.2) years were commenced on HCL. Ten (14%) discontinued HCL use, with 60% discontinuing within the first 6 months. Glycaemic outcomes were analysed in 52 children. Time spent in closed loop was 78 (21) % at 4 weeks, declined to 69 (28) % at 3 months (*P* = 0.037) and 63 (34) % at 6 months (*P* = 0.001). The mean %TIR increased from 59.8 at baseline to 67.6 at 3 months and 65.6 at 6 months with a mean adjusted difference of 7.8% points [95% CI 3.6, 11.9] and 5.5% points [95% CI 1.4, 9.5], respectively. There was a reduction in time > 10 mmol/L and time < 3.9 mmol/L from baseline to 6 months. Although families faced challenges with technology, better glucose control with reduced glycaemic fluctuations were reported.

**Conclusions:**

HCL therapy is associated with improved glycaemia; however, adequate support and education are required for best outcomes.

Insulin pumps and continuous glucose monitoring (CGM) systems are standard management strategies in type 1 diabetes (T1D). Further advances in diabetes technology have led to the incorporation of glucose‐responsive algorithms which utilise sensor glucose data to adjust insulin delivery. These are designed to reduce the need for user decisions/interventions and improve clinical outcomes. The first such system to receive regulatory approval was the Medtronic MiniMed™ 670G insulin pump which can function in two modes: ‘manual mode’ as standard pump therapy or in hybrid closed loop (HCL) as ‘auto mode’. In auto mode, the background basal insulin is automated based on frequently updated sensor glucose levels, while user‐directed input is required to deliver insulin for meals and correction of high glucose levels. As this is semi‐automated, it is a hybrid closed‐loop system.

A 3‐month trial of Medtronic 670G HCL in youth and adults with T1D reported safety and improved glycaemic outcomes which led to regulatory approval.^1^, ^2^ A retrospective analysis of 3‐month real‐world data^3^ including those from 6 months of HCL from individual paediatric^4^, ^5^, ^6^ and adult diabetes^7^ centres in Colorado confirmed glycaemic improvements in HCL users. However, of significance is a high rate of discontinuation by 6 months in youth commenced on HCL.^4^ High rates of discontinuation were also reported from a 12‐month observational study in children and adults,^8^ highlighting the need for ongoing evaluation of outcomes, and barriers to optimal system use. Youth with higher HbA1c appear to be at greater risk of ceasing HCL therapy given the high demands required to use the system as reported by patients.^9^ More recently, a 6‐month multicentre randomised control trial in 135 adolescents in Australia confirmed improvements in glycaemia compared to conventional insulin therapy and reported an increase in time in range (TIR 3.9 to 10 mmol/L) of 6.7%, corresponding to an extra hour in target glucose range per day. The study also reported an improvement in diabetes‐specific quality of life and improved treatment satisfaction with HCL.^10^ The results of this trial provide robust scientific evidence for the use of HCL in the management of youth with T1D. However, it is important to examine whether these promising outcomes are achievable in the real‐world following the roll‐out of these systems in clinical practice.

The Medtronic MiniMed™ 670G insulin pump received Therapeutics Goods Administration approval in Australia in late 2018 for individuals with T1D 7 years of age and older and is funded *via* private health insurance. The Guardian G3 sensor (required for HCL use) was subsidised by National Diabetes Service Scheme in April 2019 and is available to youth and adolescents ≤21 years of age, under the Australian Federal Government initiative that commenced in 2017. HCL therapy was introduced into clinical practice following the establishment of a structured pathway which facilitated the roll‐out of the system in our centre, but there is no real‐world data from Australia since the system became available. The objectives of our study were, first, to evaluate clinical outcomes in children and adolescents commenced on HCL therapy and, second, to collect and report the real‐life experiences of families with children using the system.

## Materials and Methods

### Study design

**Fig. 1:**
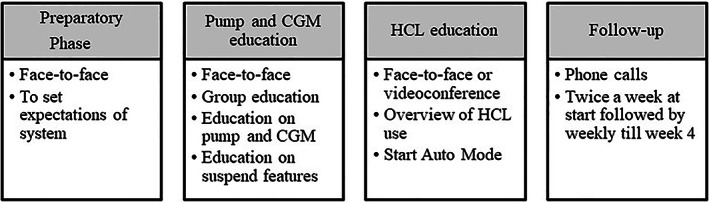
Clinical pathway of hybrid closed‐loop (HCL) start.

The study was conducted at Perth Children's Hospital; the tertiary paediatric diabetes centre in Western Australia and received project governance approval according to institutional procedures for Quality Improvement projects (project approval number 35015). Children and adolescents with T1D on a Medtronic™ 670G insulin pump and commenced on HCL in clinic were eligible and included in the analysis. The clinical pathway (Fig. 1) to commence children on closed‐loop therapy was established at our centre. There was a formal education of HCPs by the diabetes technology nurse. HCL start occurred after ensuring optimisation of pump settings in manual mode. The pathway involved regular reviews of the CareLink™ reports with families in the first 4 weeks of the closed loop start, the frequency of reviews individualised as per the need. Prospective pump data collection occurred with every clinic review. All families are advised to upload the pump prior to every clinic visit as it provides an overview of pump and CGM data to facilitate education and review.

### Data collection

Participant demographics and clinical data from the clinic cohort attending between April 2019 (coinciding with the start of HCL in clinic) and July 2021 were obtained from the Western Australian Children's Diabetes Database, a prospective register of demographic and clinical data following routine quarterly clinic visits. Pump and sensor data were collected from CareLink™ software for CGM metrics. The measured CGM metrics included 2‐week report of percentage of sensor wear, percentage of time spent in closed loop, percentage of time in range TIR (3.9–10 mmol/L), percentage of time in hypoglycaemia (<3.9 mmol/L, < 3.0 mmol/L) and percentage of time in hyperglycaemia (>10 mmol/L, >13.9 mmol/L) collected at baseline (14 days pre‐auto mode), 4 weeks, 3 months and 6 months. Participants who had baseline CGM data before start of auto mode and a minimum of 3 months data after start of auto mode, irrespective of auto mode use were included in analysis of glycaemic data. HbA1c, measured at routine clinic visits, with point‐of‐care DCA Analyser was obtained at baseline and approximately after three and 6 months after starting HCL.

### User experience: Families with T1D

To gather the user experiences of families, the technology clinical team designed questionnaires which were administered through a web‐based secure online platform to parents of children with T1D. The questionnaire was sent to the parent and the parent or the child, if deemed competent by the parent, was invited to complete the questionnaire. The data collection focussed on pathway of HCL start and the most and least disliked features of the system. Those who had discontinued HCL were encouraged to report reasons for discontinuation.^9^

### Statistics

Descriptive statistics are reported as mean (SD) or frequencies (%). Continuous variables were compared using *t* tests. Linear mixed‐effects models with time as categorical variable and random intercept for subject was used for analysis of CGM metrics to account for repeated measures within a participant and missing data. Marginal means were calculated and pair‐wise comparisons conducted between time points. The response rate of the questionnaires sent to families was obtained.

## Results

Seventy‐one children (52% males), with mean (SD) age of 12.2 ± 3.2 years with diabetes duration of 5.5 ± 3.6 years and on insulin pump therapy for 3.3 ± 3.1 years commenced HCL. Ninety‐seven percent of the cohort had experience with CGM prior to start of HCL. 56% were on Dexcom G5 CGM and the rest were on either Medtronic G2 (29.6%), Medtronic G3 (6%) and Medtronic connect (4.2%).

### Participant use of auto mode and glycaemia measures

Six children discontinued auto mode within the first 6 months of use (three discontinued in the first 3 months). After 6 months, additional four children discontinued use of the system. There was no difference in baseline glycaemic control (HbA1c) in those who discontinued HCL technology to those who continued. (*P* = 0.189).

Glycaemic outcomes were analysed for the 52 children who were commenced on HCL in clinics and had CGM data prior to auto mode start and at 3 months. Nine participants with no baseline CGM data, four participants with no CGM data at 3 months and six adolescents with prior HCL experience through research participation were excluded.

The frequency of CGM use was 80 (18) % at baseline and 85 (13) % at 4 weeks after auto mode start (*P* = 0.033). Frequency of CGM use at 3 months [83 (14) %] and 6 months [79 (20) %] was not different from baseline (*P* > 0.05). At 4 weeks, the percentage auto mode (closed loop) duration was 78 (21) % while it declined to 69 (28) % at 3 months (*P* = 0.037) and 63 (34) % at 6 months (*P* = 0.001). The percentage of auto mode duration was not significantly different between 3 and 6 months (*P* = 0.450).

The glycaemic outcomes for various glucose ranges are shown in Table 1. The mean percentage of TIR increased from 59.8 at baseline to 67.6 at 3 months with a mean adjusted difference of 7.8% points (*P* < 0.001) and to 65.6 at 6 months with a mean adjusted difference of 5.5% points (*P* = 0.009). Although there was a reduction in TIR between 4 weeks and 6 months (−2.8%; *P* = 0.013), the reduction was not significant between 3 and 6 months (−2.3%; *P* = 0.095). There was also a reduction in time spent in hyperglycaemia >10 mmol/L and time spent <3.9 mmol/L from baseline to 6 months, although there was no change in time spent in hyperglycaemia >13.9 mmol/L and time < 3.0 mmol/L. Baseline HbA1c was 7.6 (1.1)% at the start of auto mode (*n* = 59). Mean HbA1c was 7.2 (0.7) % at 3 months (*n* = 43) and 7.6 (1.0) % at 6 months (*n* = 37) after HCL start.

**Table 1:** Continuous glucose monitoring (CGM) metrics with hybrid closed‐loop (HCL) use

	Baseline	4 weeks	3 months	6 months	4 weeks – baseline		3 months‐baseline		6 months‐baseline	
CGM metrics	*n* = 52	*n* = 52	*n* = 52	*n* = 45	Difference in means	*p* value	Difference in means	*p* value	Difference in means	*p* value
TIR 3.9–10 mmol/L	59.8 (16.4)	**68.1 (9.0)**	67.6 (10)	65.6 (9.5)	8.3 (4,5,12.1)	0.000	7.8 (3.6,11.9)	0.000	5.5 (1.4,9.5)	0.009
Time > 13.9 mmol/L	10.7 (9.2)	7.0 (4.7)	8.5 (6.1)	9.1 (6.0)	−3.8 (−6.0,−1.5)	0.001	−2.3 (−4.7,0.12)	0.062	−0.9 (−3.4, 1.6)	0.265
Time > 10.0 mmol/L	26.1 (10.4)	**22.0 (5.7)**	21.0 (5.7)	22.7 (4.9)	−4.3 (−7.1,‐1.6)	0.002	−5.1 (−7.9,‐2.2)	0.001	−3.4 (−6.2,‐0.6)	0.017
Time < 3.9 mmol/L	2.7 (2.2)	2.5 (1.8)	2.3 (1.3)	1.8 (1.1)	−0.2 (−0.8,0.5)	0.560	−0.4 (−1.0,0.2)	0.165	−0.8 (−1.4,−0.2)	0.008
Time < 3.0 mmol/L	0.9 (1.3)	0.7 (0.9)	0.7 (0.8)	0.7 (0.9)	‐0.2 (−0.5,0.2)	0.400	−0.1 (−0.4,0.2)	0.490	−0.1 (−0.5,0.3)	0.551

Values in mean (SD) and median (95% CI).

### User experiences

### Responses from families

Questionnaire was sent to the first 50 families with children commenced on HCL therapy. Thirty‐eight families responded, with 76% responses completed by parents (*n* = 29). Both parent and child with T1D responded in 18% (*n* = 7) and the child only in 5% (*n* = 2). All respondents preferred to have diabetes technology education alternate between face‐to‐face and telehealth appointments. 39% of families reported ≥4 contacts while 37% reported 2–3 contacts from the time of auto mode start with the diabetes team (face‐to‐face, telehealth or email). 84% (*n* = 32/38) reported improved glycaemic control with auto mode use. Table 2 provides a list of the most liked and least liked features of auto mode. More than half of the participants reported that they liked the HCL function as the system improved glucose levels, especially overnight with less glucose fluctuation. However, frequent exits out of auto mode, the inability to monitor the child remotely, and sleep disruption with alarms ranked high on the list of least liked features of the system. Five respondents had discontinued HCL. The major contributors to HCL discontinuation were the frequency of alarms/alerts, device‐related sleep disturbance, the workload to use the device and impact on daily life.

**Table 2:** Most liked and least liked features of auto mode by families

Most liked features of auto mode	Least liked features of auto mode
Better glucose control (25)	Frequent exits out of auto mode (12)
Better control overnight (23)	Loss of ability to follow glucose levels by parent/caregiver (12)
Less fluctuations of glucose levels (21)	Sleep disrupted with alarms (10)
General ease of use (19)	No flexibility to adjust target glucose (9)
Fewer low glucose levels (15)	Sensor is inaccurate (5), too many false alerts (5)
Screen/physical display (9)	Glucose levels did not improve or worsened as much as I have been led to believe (5)
Technical features (3)	Managing pump in auto mode function requires too much work (1)

Numbers in brackets represent the number of responses in each category.

## Discussion

Globally, targets of glycaemic control are not achieved in people with T1D and hence it is important to review the outcomes, especially with newer technologies introduced into clinical care. This study comprehensively examined the real‐world glycaemic outcomes in youth with T1D. The TIR in this cohort improved from 59.8% at baseline to 65.6% at 6 months. This magnitude of improvement is similar to the 6.7% difference between HCL and standard therapy at the end of the 6‐month RCT in Australian youth with T1D.^10^ Furthermore, Berget *et al* in a real‐word study in youth reported an improvement in TIR from 50.7% at baseline to 56.9% at 6 months.^4^ A 5% increase in TIR equates to an additional 1 hour a day in target glucose range and is clinically significant^11^; every 5% increase in TIR is associated with reduction in risk of retinopathy by 28% and of microalbuminuria by 18%.^12^ In our study, the improvement in TIR with HCL was also associated with a reduction in hypoglycaemia <3.9 mmol/L and hyperglycaemia >10.0 mmol/L and corroborates the efficacy of HCL in improving glycaemic outcomes. However, there was no difference in time > 13.9 mmol/L and time < 3.0 mmol/L. This suggests Medtronic 670G HCL is effective in reducing mild hyperglycaemia but not the more pronounced hyperglycaemia, which more often than not, result from missed meal boluses. Encouraging pre‐meal boluses is an important step to reduce post prandial hyperglycaemia. Medtronic 670G was the first commercial version and was hence conservative, designed to protect against hypoglycaemia. However, time < 3.0 mmol/L was minimal at baseline in our cohort and did not reduce further with HCL. Mean HbA1c was identical at baseline and 6‐month visit, however, HbA1c data were not available in the entire sample as clinic visits were a hybrid of face‐to‐face and telehealth. Studies with HCL system have shown a 0.3% reduction in A1c with 6‐months use which is broadly concurrent with improvement in TIR found in this study.^4^, ^10^

The frequency of alarms/alerts, exits out of closed loop, device‐related sleep disturbances and inability to lower target glucose levels were recurrent challenges voiced by families. These barriers are consistent with other paediatric and adult studies^4^, ^8^ and highlight the importance of addressing these challenges and anticipated problems prior to HCL start to ensure optimal use of the auto mode function. In our study, most children were on sensors prior to commencement of HCL with the ability for remote monitoring in real‐time by caregivers. The inability to follow their child's glucose levels in real‐time with 670G HCL was a concern for parents. This has since then been rectified with the ability to follow in real‐time available in the current 770G system. Importantly, some of the challenges with frequent exits and adjustable targets are addressed with newly approved iterations of the algorithm available in the Medtronic 780G system.^13^ More stability of the algorithm with fewer exits from auto mode, auto‐correction boluses, lower programmable target glucose levels and Bluetooth connectivity enabling real‐time remote follow functionality may all contribute to reduced burden and improved glycaemic outcomes. Next‐generation advanced hybrid closed‐loop systems are also now commercially available in the Control IQ technology^14^ and Omnipod 5.^15^

In patients on Medtronic 670G HCL, encouraging sensor wear, educating on appropriate times for calibration, addressing the reasons from auto mode exit and highlighting the need for pre‐meal boluses for optimal functioning of the system remain the key parameters in gaining the benefits of the system. While most of the review revolves around patient behaviour, due consideration should be given to factors that can be modified by the HCP. A key adjustable variable is the optimization of carbohydrate‐based bolus dosing prior to commencement of HCL and review of these settings at every review.^16^ Youth using HCL therapy generally require insulin: carbohydrate ratio approximately 15% more aggressive than in standard manual mode due to insulin feedback mechanisms to enhance auto mode safety. A review of the autobasal also directs the appropriateness of the basal setting in manual mode. In summary, all insulin pump settings should be reviewed as whenever the pump exits from auto mode into manual mode, insulin delivery will be dependent on the preprogrammed basal and bolus insulin delivery.

Newer technological advancements need constant upskilling of staff and this can be challenging for HCPs planning to commence and support their patients on these systems. In an attempt to help harmonise and simplify this clinical workflow, the CARES paradigm (Calculate, Adjust, Revert, Educate, Sensor/Share)^17^ has been proposed as a framework.

**Fig. 2:**
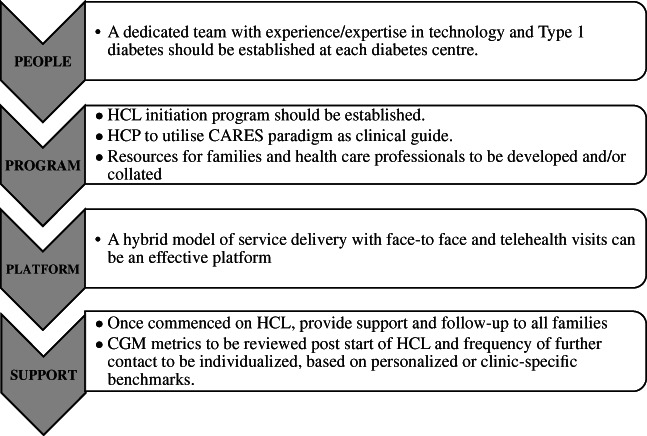
Hybrid closed‐loop (HCL) initiation program.

It is also important to ensure adequate support for families starting new diabetes technology. A dedicated team with expertise in technology and T1D should be established at each diabetes centre. A clinical pathway should be established and will need to be individualised at each centre, accounting for local demographics, clinic models, resource availability and patient characteristics. As we have shown here, families preferred a hybrid model of service delivery incorporating face‐to‐face consults and telehealth. Education and adequate support with follow‐up are important with roll‐out of new technology and models of care therefore need to be individualised. A proposed framework is presented in Figure 2.

There are strengths and limitations of this study. The major strength is the real‐world data on glycaemic outcomes alongside stakeholder experiences with HCL. However, this was a single‐centre study on a first‐generation HCL system, data on glycaemic outcomes were collected retrospectively and auto mode data were not available for the entire cohort. This study was performed during the Covid‐19 pandemic, and thus some HbA1c data were missing due to a reduction in face‐to‐face visits and thus could not be analysed. The questionnaire used for the study was designed for purpose by the clinical team but not externally validated. However, response rates were high.

Technology is fast evolving and newer therapies will be available. It is important to review these outcomes in research trials and clinical practice. The Australasian Diabetes Data Network (ADDN) is established as a national T1D registry and provides an opportunity to benchmark and evaluate these real‐life outcomes on a larger sample across the nation, in both children and adults.^18^ Centralised data collection of relevant CGM metrics will be required to inform these outcomes on an ongoing basis.

In conclusion, improved glycaemic metrics are associated with Medtronic 670G HCL therapy for youth with T1D after 6 months of real‐world use. Newer closed‐loop systems with more aggressive algorithms and fewer requirements for user input have potential to further improve clinical outcomes and acceptance while reducing user burden. A structured framework for commencement of new diabetes technology is presented here and was generally favourably received by families.

## Supporting information


**Appendix S1.** Questionnaire to families commenced on Medtronic 670G HCL.
