# Supplementary material for: Evaluation of real‐life clinical outcomes in Australian youth with type 1 diabetes on hybrid closed‐loop therapy: A retrospective study
Source: J Paediatr Child Health. 2022 May 31;58(9):1578–83. doi: 10.1111/jpc.16043 (PMC9545883; doi:10.1111/jpc.16043)
Supplement: Supplementary file 1 — Appendix S1. Questionnaire to families commenced on Medtronic 670G HCL. [file JPC-58-1578-s001.docx]

**Questionnaire to families commenced on Medtronic 670G HCL**

1. Who is answering the questionnaire?

- Parent/carer
- Child with diabetes
- Both

2. How old is the child with Type 1 diabetes? …years …months

3. In the first two months after you started Smart Guard, how many times did you have contact with the diabetes team? (this can include in person review, and contact by phone or by email)?

a. 0 b. 1 c. 2 d. 3 e. 4 f. > 4

4. How satisfied are you with the delivery of service for Smart Guard and follow-

up provided by the Diabetes nurse educators?

- Very dissatisfied
- Dissatisfied
- Neither dissatisfied or satisfied
- Satisfied
- Very Satisfied

If very dissatisfied/dissatisfied/ neither dissatisfied or satisfied how would you like us

to improve our service for Auto Mode pump start and follow-up? **Free Text**

5. Are you still using the Smart Guard function?

- Yes (if Yes will continue to answer the rest of the questions and will not answer 10)
- No (if no go to question 10)

6. Will you continue to use the Smart Guard function?

- Yes
- No
- Maybe

7. How satisfied are you with the Smart Guard function?

- Very dissatisfied
- Dissatisfied
- Neither dissatisfied or satisfied
- Satisfied
- Very Satisfied

8. What do you like MOST about the Smart Guard function of the pump?

Tick all that apply

- Better control
- Less fluctuations of glucose levels
- General ease of use
- Technical features
- Screen/physical display
- Fewer low glucose alerts
- Better sleep at night
- None of the above
- Other comments Free text

9. What do you like LEAST about the Smart Guard function of the pump? Tick all that

apply

- Sensor is inaccurate
- Too many alarms
- Too many false alerts
- Exits out of Smart Guard
- Glucose control has worsened since start of Smart Guard
- Do not have flexibility to do adjustments (to lower my target glucose level)
- Have to give up on my previous sensor
- Managing pump in Smart Guard function requires too much work
- Glucose levels didn't improve as much as I'd hoped/been led to believe
- None of the above
- Other comments Free text

If No (Question 5)

10. Move the slider to indicate how much each of the following affected your decision to discontinue the automode function.

Not a burden Major burden

0------------------------------------------------------------------------------------------10

Hassel of wearing devices all the time

How diabetes devices look on body

How diabetes devices feel on body (e.g. pain, skin rashes, bulkiness, etc)

Feeling nervous that the device might not work

Feeling like the device is interfering with a good night’s sleep

Feeling like the device is not helping with glucose levels

Too busy to use the device

Takes too much time to make it work

Too many alarms/alerts

Feeling like I can’t control insulin settings

Feeling like the device interferes with daily life

Not understanding what to do with the information or features of the device

Causes stress and fighting with family

Not enough support from diabetes care team for using the device

Feeling like there are better devices for handling diabetes
